# Genetic divergence of enterovirus D68 in China and the United States

**DOI:** 10.1038/srep27800

**Published:** 2016-06-09

**Authors:** Zichun Xiang, Zhengde Xie, Lulu Liu, Lili Ren, Yan Xiao, Gláucia Paranhos-Baccalà, Jianwei Wang

**Affiliations:** 1 MOH Key Laboratory of Systems Biology of Pathogens and Christophe Mérieux Laboratory, IPB, CAMS-Fondation Mérieux, Institute of Pathogen Biology (IPB), Chinese Academy of Medical Sciences (CAMS) & Peking Union Medical College, Beijing, People’s Republic of China, Beijing 100730, P. R. China; 2 Collaborative Innovation Center for Diagnosis and Treatment of Infectious Diseases, Hangzhou 310003, P. R. China; 3 Beijing Children’s Hospital Affiliated to Capital University of Medical Sciences, Beijing 100045, P.R.China; 4 Fondation Mérieux, 69365 Lyon, France

## Abstract

The largest outbreak of human enterovirus 68 (EV-D68) infections associated with severe respiratory illness and neurological complications emerged from the United States in 2014. China reported the circulation of EV-D68 since 2006, but these cases were sporadic and did not display neurological symptoms. Yet viral determinants responsible for the difference in prevalence between China and the U.S. were not clear. We analyzed the genome of 64 reported Chinese EV-D68 strains and found that genogroup replacement has occurred in China since 2006. The six coding mutations (M291T, V341A, T860N, D927N, S1108G and R2005K) associated with neurovirulence reported in American strains were not found in Chinese strains. Moreover, 2014 Chinese strains had a unique R220A mutation in the puff region of VP2 while R220E mutation occurred in other strains. Like other enteroviruses, the loop sequences of the domain X and Y in the 3′-UTR of the Chinese strains are complementary. However, the X loop sequences of the 2014 American strains were not complementary but identical to Y loop sequences. These results indicate that different EV-D68 strains circulated in China and America and the mutations might be responsible for different prevalence. Our findings also provide new evidence for the sequence diversity of EV-D68.

Since its discovery in 1962^1^, Enterovirus D68 (EV-D68) has historically been a rarely reported virus linked to respiratory disease. However, EV-D68 infections have notably increased worldwide in recent years^2^,^3^,^4^,^5^,^6^,^7^,^8^,^9^. In particular, EV-D68 outbreaks in the United States in 2014 have raised concerns of a pandemic^7^,^8^,^9^. Similarly, between 2006 and 2014 EV-D68 infections in China were also detected in children and adults with respiratory infections^4^,^10^,^11^,^12^,^13^. In contrast to the symptoms caused by EV-D68 and the prevalence observed in the United States, none of the cases in China had nervous system dysfunction and no notable outbreak was reported. Yet little is known about the viral factors that have led to the difference in EV-D68 prevalence.

To control emergent EV-D68 epidemics, intensive investigations on the epidemiological and genomic characteristics are needed to identify viral determinants responsible for transmission. EV-D68 belongs to Enterovirus D species within the genus *Enterovirus* in the family *Picornaviridae*. EV-D68 is a non-enveloped, single-stranded RNA virus consisting of the genome-linked protein VPg at the 5′ end, a highly structured 5′-untranslated region (UTR), a single open reading frame coding for a polyprotein, a 3′-UTR, and a poly (A) tail. The polyprotein is cleaved into 4 viral capsid proteins VP1—VP4 and 7 non-structural proteins involved in protein processing and genome replication, including 2A—2C and 3A—3D by its proteases 2A and 3C^14^. The VP1 gene is used to classify EVs into different serotypes or genotypes^15^. VP1, VP2 and VP3 are located on the external surface of the capsid shell and harbor the neutralizing immunogen sites^16^. The 3′-UTR consists of 68 nucleotides and contains a secondary pseudoknot structure that has been implicated in controlling viral RNA synthesis^17^.

To understand the prevalence of EV-D68 in China, we analyzed 64 Chinese EV-D68 sequences including 54 sequences, which were previously identified^4^,^10^,^11^,^12^,^13^, two sequences submitted directly to Genbank, and eight sequences that were detected from children hospitalized in Beijing Children’s Hospital between 2007 and 2014. Our results revealed sequence differences in the 3′-UTR and puff region of VP2 as well as six coding polymorphisms that are associated with neurovirulence^7^.

## Methods

### Sequences and phylogenetic analysis

Fifty-six partial or complete sequences of Chinese EV-D68 VP1 genes available in GenBank (http://www.ncbi.nlm.nih.gov) were retrieved on December 1, 2015. In addition, we also detected nasopharyngeal aspirates that were collected from 4,307 pediatric patients (2,671 boys, age range 0.3–168 months; median: 15.3 months) who had lower respiratory tract infections (RTIs) when they were admitted to the Beijing Children’s Hospital from March 2007 to December 2014. All samples were collected after obtaining informed consent from the individual’s guardians. This study was approved by the ethical review committee of the Institute of Pathogen Biology, Chinese Academy of Medical Sciences. The methods were carried out in accordance with the approved guidelines. To detect EVs, we amplified 350–400 bp of the VP1 gene by reverse transcription PCR^15^ after nucleic acid extraction from nasopharyngeal aspirates using the NucliSens easyMAG^TM^ platform (bioMérieux, Marcy l’Etoile, France).

In these pediatric patients, 8 children (5 boys, age range 1–122 months; median: 47.8 months) were EV-D68 positive (GenBank accession nos. KT285479–KT285485, KF726085). We designed primers based on the sequence of Fermon strain, which is the prototype EV-D68. We amplified 3 complete genome sequences and 5 complete VP1 sequences from nasopharyngeal aspirates directly. Among them, one complete genome sequence (KF726085) was submitted to GeneBank on October 8, 2013. The Chinese EV-D68 sequences submitted to GenBank have different lengths (343–7348bp). To understand the prevalence of EV-D68 in China, we used the 338 bp fragment of VP1 gene to construct the phylogeny to get data as much as possible. The Maximum Likelihood tree of the partial EV-D68 VP1 gene [338-bp fragments, which corresponds to the locations of nt 2518–2855 of the EV-D68 prototype strain (GenBank accession no. AY426531)] was constructed using the Tamura-Nei model with a Molecular Evolutionary Genetics Analysis (MEGA) software version 5.10 (www.megasoftware.net)^18^.

As of January 8, 2016, there were 112 (including KF726085) complete or nearly complete genome sequences of EV-D68 in Genbank, but only 39 had the entire 3′-UTR. Of the 39 complete sequences, 14 were submitted from China and and 20 were submitted from USA. Together with the two complete sequences (KT285484, KT285485) amplified in this study, we analyzed the 3′-UTR characteristics of 41 sequences in total using the BioEdit software.

### Evolutionary analysis

The evolution rates of different Chinese EV-D68 genogroupes were determined based on partial VP1 gene sequence (338 bp) data by the Bayesian Markov Chain Monte Carlo (Bayesian MCMC) method implemented in BEAST (v1.8.1), using a relaxed molecular clock (uncorrelated lognormal-distributed model)^19^. The best substitution models were selected using Modeltest (version 3.7) according to Akaike information criterion (AIC)^20^. Each Bayesian MCMC analysis was run for 20 million states and sampled every 5,000 states. Posterior probabilities were calculated using Tracer (version 1.5). Bayesian skyline plots for EV-D68 genogroups were estimated to depict the relative viral genetic diversity over time.

## Results

### Temporal and genogroup distribution of EV-D68 in China (2006–2014)

**Figure 1:**
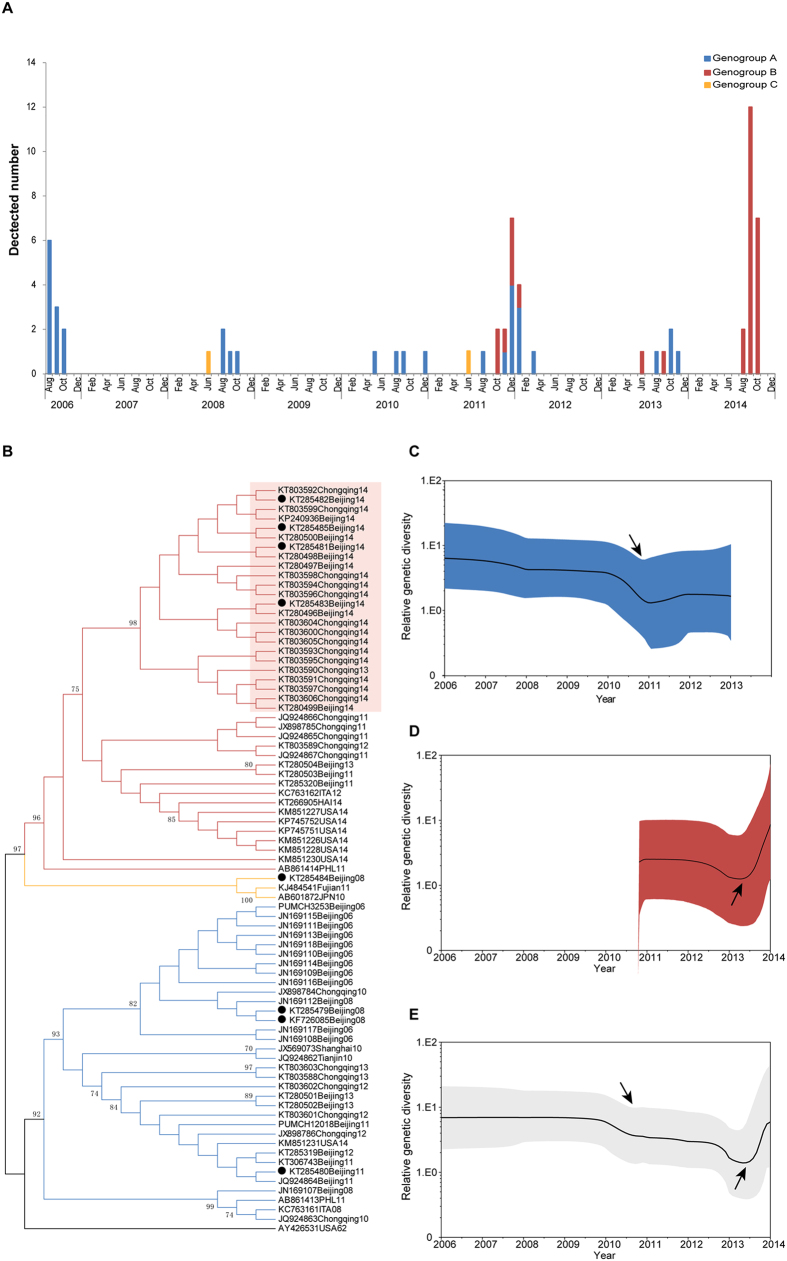
Temporal distribution and phylogenetic analysis of EV-D68 infections in the People’s Republic of China, August 2006-December 2014. Genogroup A is indicated in blue and Genogroup B is indicated in red. (**A**) Monthly distribution of EV-D68 detected in China. Numbers of EV-D68 cases in each month during the study period are shown on the right-side y-axis. (**B**) Phylogenetic analysis of EV-D68 according to partial viral protein 1 (VP1) (338-bp fragments, which correspond to the locations of nt 2518–2855 of the EV-D68 prototype strain (GenBank accession no. AY426531)) nucleotide sequences. The Maximum Likelihood tree was generated with 500 bootstrap replicates using the Tamura-Nei model. The strains identified in this study are indicated by black solid circles. Chinese strains which had T207 and R220A substitutions are shaded. (**C–E**) Relative genetic diversity dynamics of Chinese EV-D68 strains using sequences of 64 EV-D68 partial VP1 genes obtained from 2006 to 2014. Bayesian skyline plot estimates depicting the past genetic diversity dynamics of EV-D68 were generated using Bayesian Markov chain Monte Carlo analysis implemented in Beast 1.8.1. The medians (g) are represented by the black lines and 95% high posterior densities are shown in the colored regions. (**C**) Genetic diversity of Genogroup A was determined using the HKY+G substitution model. (**D**) Genetic diversity of Genogroup B was determined using the TrN+G substitution model. (**E**) The total genetic diversity was determined using the TrN+G substitution model.

A total of 64 partial VP1 gene sequences from China, including 56 sequences that were obtained from Genbank and eight sequences obtained from pediatric clinical samples in this study, were used for analysis (Fig. 1). These 64 EV-D68 sequences were detected from 64 RTIs cases which distributed in 5 different geographical locations (Beijing, Tianjin, Shanghai, Fujian and Chongqing) indicating a wide distribution of EV-D68 in China. There were three periods of heightened EV-D68 activity in China during the study period: August–October 2006, October–December 2011, and August–October 2014 (Fig. 1A).

Based on the VP1 nucleotide sequences, the circulating strains of EV-D68 were classified into three genogroups: A, B and C^3^. Of the 64 sequences, we found that the EV-D68 viruses belong to genogroup A (n = 33; 51.56%), B (n = 29; 45.31%) and C (n = 2; 3.13%). From 2006 to 2014, the circulation of EV-D68 occurred primarily in summer and autumn, although their prevalence in 2011 extended into the spring of 2012. Before August 2011, the strains of genogroup A were predominant (20/22) and the two strains of genogroup C only scattered in June of 2008 and 2011. The genogroup B strains emerged in October 2011 and co-circulated with those of genogroup A until November 2013 (A/B = 13/8). The strains of genogroup B contributed to the EV-D68 infections of 2014 alone (Fig. 1A, B) (N = 21). These results suggest that in China from 2006 to 2014, the dominant circulating EV-D68 strains shifted from genogroup A to genogroup B.

### Relative genetic diversity of Chinese EV-D68

Bayesian skyline plot analyses were estimated to depict the relative genetic diversity of Chinese EV-D68 in a partial VP1 gene (338 bp) over time (Fig. 1C–E). Because of the limited number of genogroup C, we only analyzed the genetic diversity of genogroups A and B. Genogroup A showed a decrease in genetic diversity in 2011 (Fig. 1C) while genogroup B showed a sharp increase in genetic diversity in 2014 (Fig. 1D). Thus, the overall dynamics of all Chinese sequences had two sharp changes in genetic diversity (Fig. 1E) due to the variation of genogroups A and B. The estimated changes in genetic diversity are consistent with epidemiological data in China (Fig. 1A). The ratio of genogroup A to total EV-D68 cases has been reduced after August 2011and the detection number of genogroup B increased from October 2011 and reached its summit in 2014.

### Coding polymorphisms of EV-D68 strains

**Figure 2:**
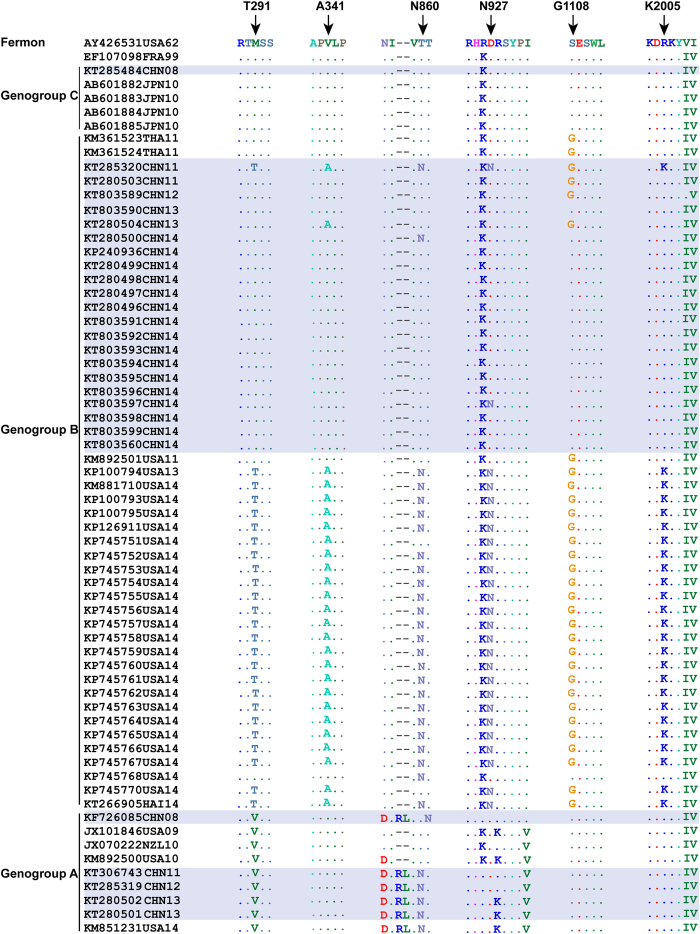
Coding polymorphisms associated with EV-D68 (Greninger clade B1^7^). Multiple sequence alignment of the six coding polymorphisms using BioEdit software version 3.3.19.0. Conserved residues are represented by dots and Chinese strains are shaded.

Greninger *et al*. recently reported that six coding polymorphisms (M291T, V341A, T860N, D927N, S1108G and R2005K) in EV-D68 might have conferred an increased propensity for neurovirulence^7^. Because none of the EV-D68 cases in China had nervous system dysfunction, so we first sought to test if EV-D68 strains from China had these six mutations. Amino acid alignment of the 41 EV-D68 strains that had complete genome sequences revealed that none of the strains in genogroup A had a coding polymorphisms above six. In genogroup B, however, all 2014 American strains had these six substitutions while only one of the Chinese strains (KT285320) had mutations at these six sites (Fig. 2).

**Figure 3:**
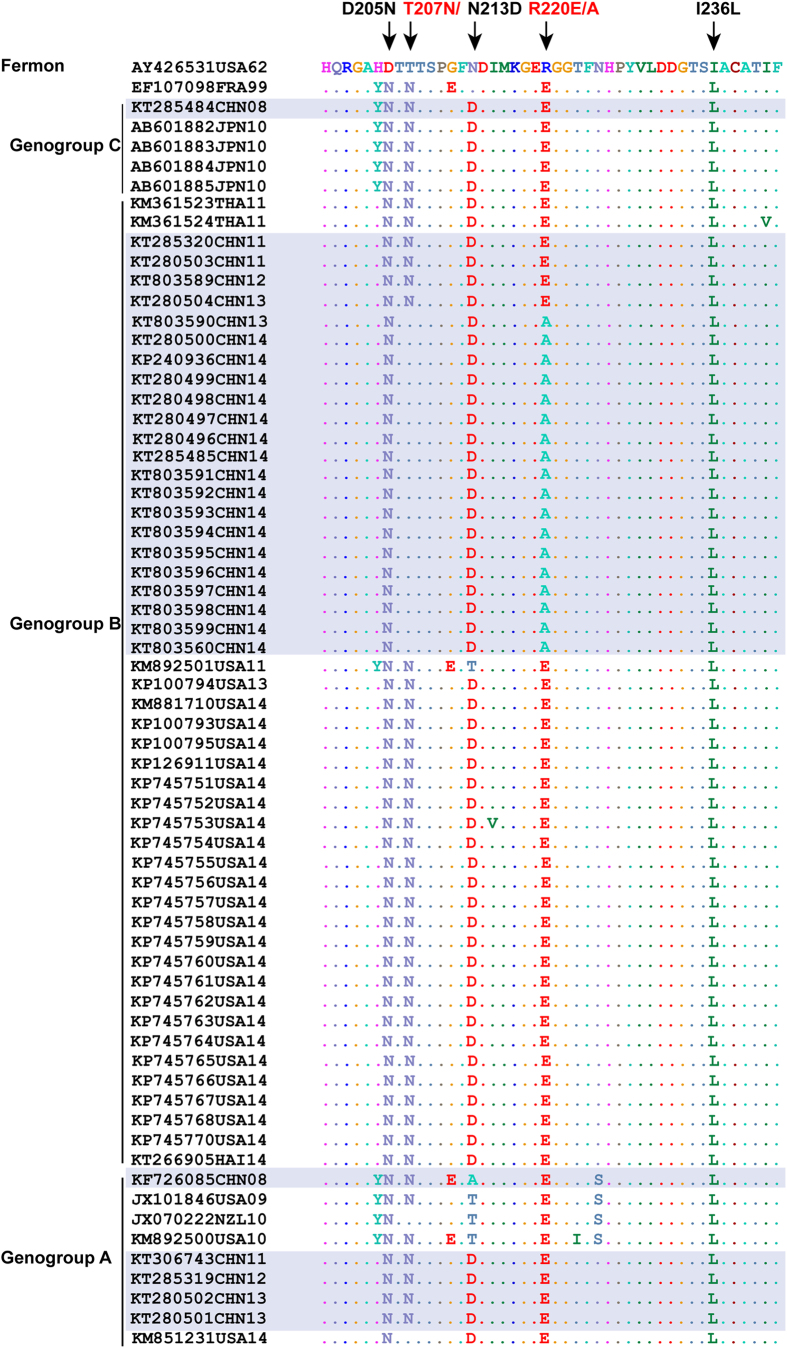
Multiple sequence alignment of puff region in VP2. Multiple sequence alignment of puff region in VP2 was performed by using BioEdit software version 3.3.19.0. Conserved residues are represented by dots and Chinese strains are shaded.

We then analyzed the puff region of all EV-D68 sequences. The puff region is known to be a major neutralization site in polioviruses and rhinoviruses^21^,^22^. In aligning amino acids to the prototype strain Fermon, our analysis showed five substitutions (D205N, T207N, N213D, R220E, and I236L) (Fig. 3). These five substitutions occurred in most of the strains of genogroup A and genogroup B. However, one 2013 Chinese strain and all 2014 Chinese strains had two notable changes: a reverse mutation at T207 and an R220A substitution. These strains form a unique branch in the VP1 phylogenetic tree (Fig. 1B shaded).

### Sequences of 3′-untranslated region (UTR) in EV-D68 strains

**Figure 4:**
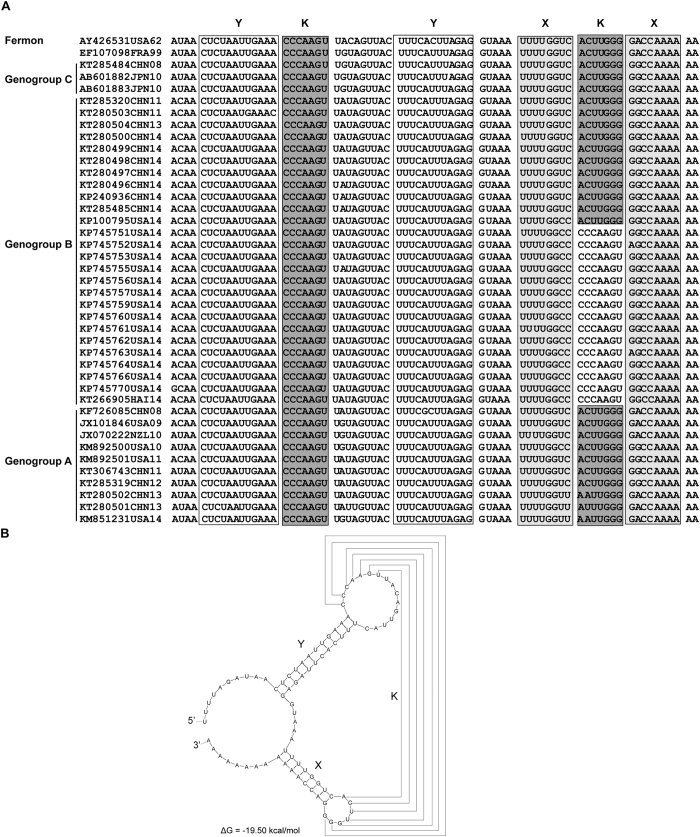
Sequence alignment and structure of the EV-D68 3′-UTR. (**A**) Multiple sequence alignment of the EV-D68 3′-UTR was performed using BioEdit software version 3.3.19.0. The X, Y, and kissing-like (K) domains are indicated in the figure. (**B**) Secondary structure of the individual domains X and Y of the EV-D68 3′-UTR. The kissing-like pseudoknot interaction is designated as connecting lines between nucleotides in loops X and Y.

The enterovirus 3′-UTR contains a pseudoknot structure formed by the folding of two stem-loop domains designated X and Y. The loop-structures of domain X and domain Y form a kissing (K) interaction by their complementary sequences^23^ (Fig. 4B). The stems of X and Y consist of 8 base pairs (bp) and 12 bps, respectively^23^. We analysized 41 EV-D68 sequences that had complete 3′-UTR. Alignment showed that the 3′-UTR of EV-D68 also contains X and Y domains and the stems of X and Y are conserved in all EV-D68 strains (Fig. 4A). Furthermore, the loop-structure sequences of domain X and domain Y were complementary in most strains including all 2014 Chinese strains and formed a K interaction (Fig. 4B). However, the X loop sequences of the available 2014 American strains and 2014 Haiti strain were not complementary but the same as Y loop sequences and could not form a K interaction (Fig. 4A).

## Discussion

In our study, we examined 64 Chinese EV-D68 sequences and found strains of genogroup A dominated in China before 2010 and strains of genogroup B predominated in 2014. Genogroup A co-circulated with genogroup B from 2011 to 2013. Accordingly, the overall dynamics of all Chinese sequences had two sharp changes in genetic diversity. Our findings provide evidence that an ongoing genogroup replacement of circulating EV-D68 occurred in China between 2006 and 2014.

Genogroup switching is common and often associated with the occurrence of new outbreaks in other EVs, e.g. CVA16 and EV71^24^. Recently, Greninger *et al*.^7^ and Huang *et al*.^25^ reported that the 2014 U.S. outbreak was caused by a new clade of EV-D68. However, the genogroup switching of EV-D68 did not cause a notable outbreak in China. Viral factors that have led to the difference in EV-D68 prevalence have yet to be elucidated.

To investigate sequences of circulating EV-D68 strains, we first compared six reported coding polymorphisms (M291T, V341A, T860N, D927N, S1108G and R2005K) of EV-D68 strains, which are associated with neurovirulence^7^. T860 and S1108 are cleavage sites of 2A^pro^ and 3C^pro^. Mutations of T860N and S1108G are considered to alter the proteases’ cleavage efficiency and increase replication and transmission rate of EV-D68^25^. Aside from the one strain reported in 2011, the six coding polymorphisms did not appear in the Chinese strains.

EV-D68 infected cases associated with neurological symptoms were also reported in other countries such as in France, United Kingdom, Italy, Norway and Australia^26^,^27^,^28^,^29^,^30^,^31^. However, the authors of these studies either only submitted partial sequences of VP1 (207–861bp), or did not provide the sequences. Thus, we cannot determine whether these EV-D68 strains possess the six mutations when associated with neurological symptoms. Greninger *et al*. note that some patients with only upper respiratory illness were infected with the identical enterovirus D68 strains as patients with neurological disease^7^. This result indicates that the six mutations are necessary but may not be sufficient to cause neurological symptoms. Experimental evidence or a larger number of observations would add weight to this hypothesis.

A single neutral asparagine-to- acidic aspartate mutation in the puff region of VP2 markedly altered the ability of CVB3 to induce myocarditis^32^. We also noted that at amino acid 220 of this region, there was an arginine-to-alanine mutation in the 2014 Chinese strains. However, the arginine-to glutamic acid mutation occurred at this site in other strains. Whether the mutation at this site contributed to the difference in virulence between Chinese strains and American strains remains to be determined.

Finally, we observed differences in the 3′UTR sequences of the 2014 American strains compared to other EV-D68 strains. Like other enteroviruses, the complementary loop-structure sequences of the domain X and Y in most EV-D68 strains form a kissing-interaction. Yet the X loop sequences of the available 2014 American strains are not complementary but rather are the same as the Y loop sequences. This change in sequence can affect the formation of the pseudoknot structure in 3′-UTR and thus the replication of the enterovirus^33^. As demonstrated by Wang *et al*., base pair mutations in the 3′-UTR of CVB3 revealed that A∙A and U∙U mutations resulted in temperature sensitive phenotypes while G∙G or C∙C mutations resulted in lethal phenotypes^34^. However, compared to the Fermon strain, all of the 2014 American strains have C∙U or C∙A mutations. We presumed that this mutation in the 3′-UTR might result in altered phenotypes. These mutations might on the one hand enhance the viral replication, and on the other hand may increase epidemiological fitness by evading the innate immune response. For instance, in dengue virus the predominant epidemic strains had A∙G and U∙C substitutions in pseudoknots of the 3′-UTR^35^. These substitutions led to increased epidemiological fitness through binding to and prevention of tripartite motif 25 (TRIM25) deubiquitylation, which is critical for reducing type-I interferon expression^35^. Whether the mutations in the 3′-UTR contributed viral factors that led to the 2014 outbreak of EV-D68 infections in the U.S. needs to be studied further by mutation experiments.

In conclusion, we provide evidence that a genogroup replacement of circulating EV-D68 occurred in China between 2006 and 2014. However, this genogroup change did not cause a notable outbreak in China unlike in the U.S. This difference might be attributed to sequences in the six coding polymorphisms associated with neurovirulence, the puff region of VP2, and the X loop sequences of the 3′-UTR. Our findings provide new evidence about the sequence diversity of EV-D68. Further study is needed to determine if these sequence diversity affected the viral pathogenicity.

## Additional Information

**How to cite this article**: Xiang, Z. *et al*. Genetic divergence of enterovirus D68 in China and the United States. *Sci. Rep.* **6**, 27800; doi: 10.1038/srep27800 (2016).
